# P25 An opportunity for antimicrobial stewardship? Assessing the diagnosis and treatment of lower urinary tract infections among inpatients aged 65 and older in a large English teaching hospital

**DOI:** 10.1093/jacamr/dlac004.024

**Published:** 2022-02-16

**Authors:** Shauna Henry, Abimbola Olusoga

**Affiliations:** Leeds Teaching Hospitals NHS Trust, Leeds, UK; Leeds Teaching Hospitals NHS Trust, Leeds, UK

## Abstract

**Background:**

Between 2015 and 2019 the rate of bacteraemias reported in the UK increased by 16.9% along with an increase of 32.5% of resistant organisms associated with bloodstream infections. This increase appears to be driven by a high number of Gram-negative bloodstream infections (GNBSI). There were 312 GNBSI within Leeds Teaching Hospital NHS Trust (LTHT) for the financial year (FY) of 2019–20 and 275 in FY 2020–21. *Escherichia coli* has been identified as the most common Gram-negative organism associated with bacteraemias. Urinary tract infections (UTIs) have been consistently highlighted as a primary source of *E. coli* infections, alongside other Gram-negative infections, therefore justifying a review of practice within LTHT regarding diagnosis and treatment of UTI in patients over the age of 65. This quality improvement work aimed to build on the foundations laid by NHS England's Commissioning for Quality and Innovations (CQUIN) scheme 2019–20, which had a focus on the treatment of UTIs in patients over the age of 65. This interventional prospective project was carried out to assess the impact of an antimicrobial stewardship (AMS) pharmacist-led review of patients prescribed antimicrobial treatment for UTIS.

**Objectives:**

To assess the impact of a pharmacist led clinical review of the diagnosis and treatment of UTIs in patients over the age of 65.

**Standards:**

Eighty percent compliance was expected in the following standards: (i) patients having symptoms of a UTI documented in their clinical notes; (ii) a urine dipstick is *not* used as part pf the diagnosis pathway; (iii) have a had a midstream urine sample sent; and (iv) if appropriate, an antibiotic prescribed that complies with NICE/Trust guidelines.

**Methods:**

A prospective study of 87 inpatients prescribed antimicrobials for lower UTI (LUTI) was conducted from September 2020 to November 2020. The study aimed to evaluate if LUTI was diagnosed in accordance with PHE guidance, treated as per Trust guidelines and the impact an AMS pharmacist interventions had on treatment. Patients were identified using a daily antimicrobial list; searching and filtering based on indication for antimicrobial prescribed. Online medical notes were reviewed for information relating to the standards, where no information could be found wards were contacted with recommendations or further questions. If a patient was identified as requiring an intervention, for example a change of treatment, this was communicated with the ward team by an appropriate route as well as documenting in the medical notes. Inclusion criteria: >65 years old, prescribed an antibiotic for the treatment of LUTI or catheter associated UTI. Exclusion criteria: <65 years old, complicated UTI, upper UTI, urosepsis.

**Results:**

Eighty-seven patients were audited against the criteria: documentation of UTI symptoms = 74%; urine dipstick was not used to diagnose UTI = 69%; urine sample requested = 82%; appropriate antimicrobial was prescribed = 76% (Figures 1 and 2). As a treatment pathway 32% (28 patients) met all four criteria required to accomplish compliance to the diagnosis and treatment of LUTI. Of the 24% of antimicrobial prescriptions deemed inappropriate common themes included: not prescribing appropriate first line antibiotic; no clear indication for antibiotics; resistant to antibiotic prescribed; and inappropriate IV antibiotic prescribing (Figure 3). After an AMS pharmacist contacted the clinical teams to discuss the 24% of inappropriate antimicrobials it resulted in 92% of the antimicrobials being amended to appropriate antimicrobial choices. Increasing appropriate antimicrobials prescribed from 76% to 99%.

**Conclusions:**

The results show that whilst most patients met some of the criteria expected to diagnose a LUTI, most did not meet all four. This warrants further quality improvement work to improve the recognition and adherence of the diagnosis and treatment of LUTIs with the aim to embed the processes across all age groups. The interventions of an AMS pharmacist positively impacted the prescribing of antimicrobials in LUTI and appears to be significant, particularly with regards to stopping inappropriate antimicrobials and recommending better treatment choices. These decisions included: promoting oral antibiotics instead of IV; recommending effective treatment based on renal impairment; acting on urine culture sensitivities; and choosing more narrow spectrum antibiotics. This provides a new potential tool for targeted AMS in relation to the treatment of LUTI within a large scale teaching hospital.

**Figure 1.**
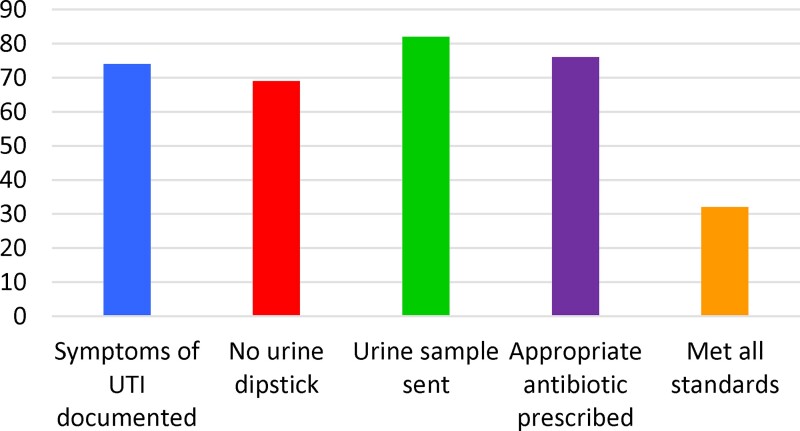
Graph showing the percentage of datasets meeting the standard.

**Figure 2.**
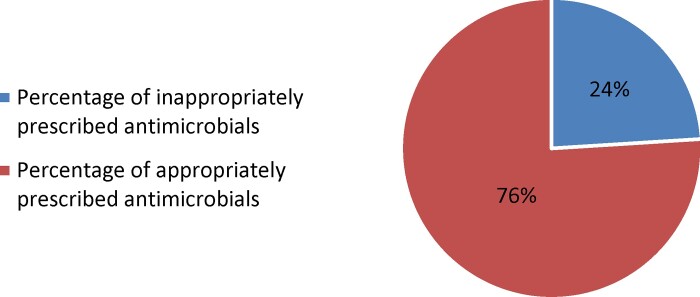
Pie chart showing the percentage of inappropriate versus appropriate antimicrobial prescriptions.

**Figure 3.**
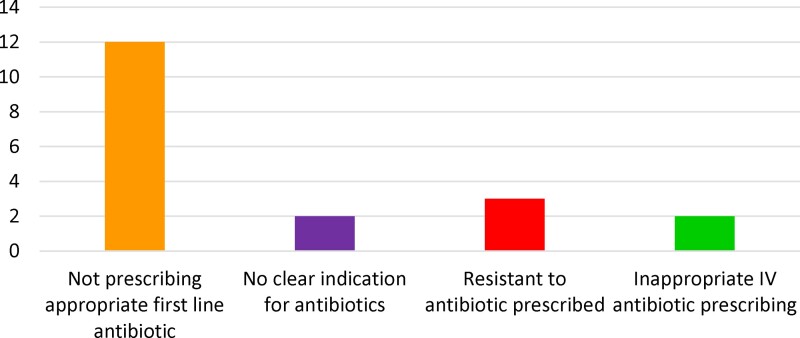
Bar chart showing the number of inappropriate antimicrobials and the reasons associated with them being inappropriate.
